# Timing of False Ring Formation in *Pinus halepensis* and *Arbutus unedo* in Southern Italy: Outlook from an Analysis of Xylogenesis and Tree-Ring Chronologies

**DOI:** 10.3389/fpls.2016.00705

**Published:** 2016-05-24

**Authors:** Veronica De Micco, Angela Balzano, Katarina Čufar, Giovanna Aronne, Jožica Gričar, Maks Merela, Giovanna Battipaglia

**Affiliations:** ^1^Department of Agricultural Sciences, University of Naples Federico II, NaplesItaly; ^2^Biotechnical Faculty, Department of Wood Science and Technology, University of Ljubljana, LjubljanaSlovenia; ^3^Slovenian Forestry Institute, LjubljanaSlovenia; ^4^Department of Environmental, Biological and Pharmaceutical Sciences and Technologies, Second University of Naples, CasertaItaly; ^5^Laboratoire Paléoenvironnements et Chronoécologie, École Pratique des Hautes Études, Université de Montpellier, MontpellierFrance

**Keywords:** cambial activity, cambial phenology, mediterranean climate, intra-annual density fluctuations, tree rings

## Abstract

Mediterranean tree rings are characterized by intra-annual density fluctuations (IADFs) due to partly climate-driven cambial activity. IADFs are used as structural signals to gain information on relations between environmental conditions and eco-physiological processes during xylogenesis, with intra-annual resolution. To reach an unbiased synchronization of the IADF position within tree rings and seasonal fluctuations in environmental conditions, it is necessary to know the timing of cambial activity and wood formation, which are species- and site-specific processes. We applied the microcoring technique to analyze xylogenesis in *Pinus halepensis* and *Arbutus unedo*. To the best of our knowledge, this is the first attempt to study xylogenesis in a hardwood species forming frequent IADFs. Both species co-occur at a site in southern Italy characterized by a Mediterranean climate. To facilitate tree-ring dating and identification of IADFs, we performed traditional dendroecological analysis. We analyzed xylogenesis during summer, which is considered a constraint for xylogenesis and a trigger for IADF formation. We followed the different phases of cell development in the current wood increment with the aim of evaluating *whether* and *which* type of IADFs were formed. We additionally analyzed the same phases again in September and in winter to verify the possible formation of IADFs in fall and whether cell production and differentiation was completed by the end of the calendar year. Both species formed the same type of IADFs (earlywood-like cells within latewood), due to temporary growth restoration triggered by rain events during the period of summer drought. At the end of the calendar year, no cells in the phases of enlargement and secondary cell wall deposition occurred. *A. unedo* was more sensitive than *P. halepensis* because IADFs were formed earlier in the season and were more frequent in the tree-ring series. The dendro-anatomical approach, combining analysis of tree-ring series and of xylogenesis, helped to detect the period of IADF formation in the two species. Results are discussed in functional terms, highlighting the environmental conditions triggering IADFs, and also in methodological terms, evaluating the applicability of xylogenesis analysis in Mediterranean woods, especially when the formation of IADFs is not uniform around the stem.

## Introduction

Tree rings are well-established climate proxies: environmental information can be extracted from dated tree-ring series by analyzing the variability in tree-ring width, earlywood and latewood widths, wood density and functional anatomical traits (e.g., Eckstein and Schmidt, 1974; Schweingruber, 1978; Eckstein et al., 1979; Tardif, 1996; Cherubini et al., 2003; Grudd, 2008; Fonti et al., 2010; Esper et al., 2012; Beeckman, 2016). The analysis of tree-ring series is relatively easily applied in plants from temperate regions characterized by a clear seasonality inducing a dormancy in cambial activity once a year (e.g., Prislan et al., 2013a). Under such conditions, each ring corresponds to one calendar year, with earlywood and latewood, respectively, linked to spring and summer climatic conditions (Fritts, 1976).

The advancement of tools of digital image analysis has raised new interest in the application of quantitative wood anatomy to tree-ring series to study a plant’s response to environmental changes (Fonti et al., 2010; von Arx and Carrer, 2014). The analysis of tree-ring series is more and more applied in various climatic regions worldwide and has a great potential to reconstruct environmental information with seasonal or intra-seasonal resolution, especially under conditions promoting an alternation of growth flushes and dormancy during the year (De Micco et al., 2016a). Within this context, wood of Mediterranean species is particularly interesting because frequent fluctuations in climatic factors exert a control on cambial activity, thus triggering the formation of intra-annual density fluctuations (IADFs) in tree rings (Cherubini et al., 2003; De Micco and Aronne, 2009; De Micco et al., 2016a). The increasing drought and changes in the frequency of precipitation and extreme events forecasted for the Mediterranean basin (IPCC Working Group I et al., 2013) will likely influence trends in cambial phenology and xylogenesis, thus the frequency and structural features of IADFs (Vieira et al., 2010). Since different species can show different sensitivities to fluctuating environmental conditions and can be differently prone to form IADFs in various environments, understanding the patterns and processes of xylem formation in response to variable environmental conditions is valuable for forecasting species growth fitness and adaptation capability (Camarero et al., 2010), which are ultimately linked to forest dynamics, biomass production and biogeochemical cycles (Cuny et al., 2015; Xia et al., 2015; Pacheco et al., 2016).

IADFs have been considered a constraint in dendrochronology until recently but they have been finally accepted as “positive anomalies” in tree rings because their analysis furnishes information on the relations between environmental conditions and eco-physiological processes during wood formation, with intra-annual resolution (Campelo et al., 2007a,b, 2013; de Luis et al., 2007, 2011a; De Micco et al., 2007, 2012, 2014; Battipaglia et al., 2010, 2014; Vieira et al., 2010; Rozas et al., 2011). In the last decade, numerous studies have analyzed IADFs in Mediterranean softwoods and hardwoods, also raising hypotheses on the factors responsible for their formation (De Micco et al., 2016a). Several classifications of IADFs have been proposed based on their position within the ring and on anatomical traits (e.g., lumen diameter and cell-wall thickness) of the xylem conduits in the IADF zone (Campelo et al., 2007a,b, 2013, 2015; Battipaglia et al., 2010, 2014; De Micco et al., 2012, 2014). In Mediterranean conifers growing at coastal sites in south-eastern Spain, the most common IADFs are classified as type-*L* (large-lumen and thin-walled earlywood-like cells within narrow-lumen and thick-walled latewood conduits), whose formation has been linked to the reactivation of cambial activity, due to favorable conditions in fall after a period of summer drought (de Luis et al., 2011a,b; Campelo et al., 2013; Novak et al., 2013a,b, 2016; Carvalho et al., 2015; Vieira et al., 2015). Type-*E* IADFs (narrow-lumen and thick-walled latewood-like cells within large-lumen and thin-walled earlywood conduits) have been described in *Pinus pinaster* growing in Italy and are considered a response to summer drought conditions inducing stomata closure (De Micco et al., 2007). Both types of IADFs have also been found in a few Mediterranean hardwoods (Campelo et al., 2007a, 2010; Battipaglia et al., 2010, 2014; De Micco et al., 2012, 2014).

Hypotheses on the reason for IADF formation derive from indirect evidence, namely correlations with climate variables (i.e., temperature and precipitation) but knowledge gaps still remain to be filled (Battipaglia et al., 2016; De Micco et al., 2016a; Zalloni et al., 2016). The formation of IADFs in Mediterranean species has been mainly linked to water availability, which affects the turgor-driven expansion of xylem cells (Sperry et al., 2006; De Micco et al., 2016a). However, to confirm such a hypothesis, the study of IADFs during their formation is needed through analysis of xylogenesis aimed at unraveling *how* and *when* wood with specific anatomical traits is formed (Vaganov et al., 2006; Camarero et al., 2010; de Luis et al., 2011b; Vieira et al., 2014; Novak et al., 2016). The analysis of xylogenesis in woods forming IADFs with high frequency is useful for achieving precise synchronization of the IADF position within the tree ring and specific environmental fluctuations triggering them. Analysis of cambial activity has been widely applied through micro-coring techniques, mostly in conifers and hardwoods growing in temperate climates (Rossi et al., 2003, 2006, 2008, 2012; Čufar et al., 2011; Prislan et al., 2013b; Gričar et al., 2014; Pérez-de-Lis et al., 2016). Analysis of xylogenesis has also recently been applied to tree rings forming IADFs in *Pinus* species (de Luis et al., 2011a; Vieira et al., 2014, 2015; Novak et al., 2016). However, to the best of our knowledge, there are no reports dealing with the study of IADF-genesis in hardwood species.

In this study, we analyzed cambial activity in a softwood and a hardwood species, *Pinus halepensis* Mill. and *Arbutus unedo* L., co-occurring at a site in southern Italy, characterized by a Mediterranean climate. The work aimed at: (1) evaluating *whether* and *which* type of IADFs were formed during summer, and (2) highlighting which weather conditions were concomitant or closely preceding IADF formation. Together with the two ecological aims, we also pursued a third methodological issue. In view of the fact that the formation of IADFs is a variable phenomenon along the stem circumference in Mediterranean woods (Cherubini et al., 2003), we aimed to verify the degree of applicability of the micro-coring technique to the two species, especially considering that *A. unedo* is a hardwood species. Thus, we applied common dendroecological techniques to analyze tree-ring chronologies and evaluate their variability within and between plants.

We performed microcore sampling and microscopy analysis of thin cross sections during the period of summer aridity (which is considered a factor limiting plant growth and predisposing the occurrence of IADFs) to detect the time of IADF formation. In order to do this, we first evaluated the onset of latewood formation during the development of tree rings with and without IADFs, then continued the analysis of xylogenesis, searching for earlywood-like cells, until we detected the formation of an IADF in the 2014 tree-ring. This was based on the hypothesis that IADFs in both species are formed during the summer months, triggered by water stress followed by the temporary restoration of growth as a consequence of favorable conditions. We therefore hypothesized that both species experience a bimodal pattern of cambial activity, as reported in Camarero et al. (2010), thus completing their ring growth by the end of the calendar year. To verify this hypothesis, we also analyzed xylogenesis at additional dates until the end of the calendar year. This helped us to verify that ring growth had been completed by the end of the calendar year, as commonly assumed in traditional dendrochronology, and to verify the possible formation of other IADFs primed by rain events in fall.

## Materials and Methods

### Species and Study Area

**FIGURE 1:**
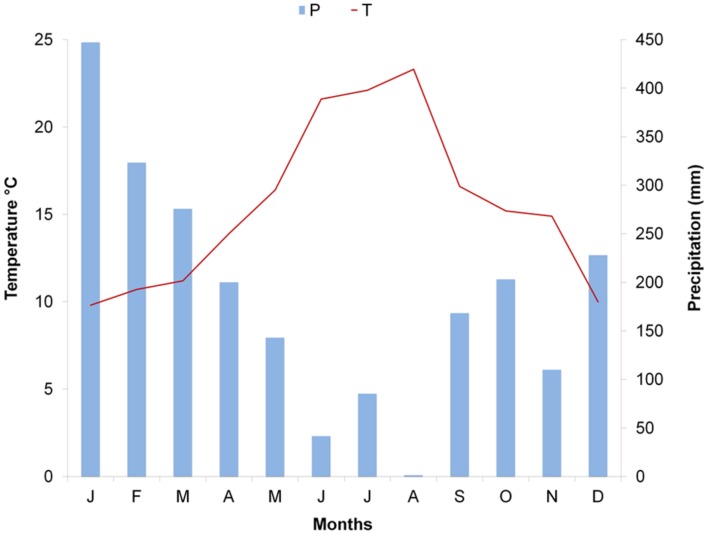
**Climatic diagram of Pimonte meteorological station: monthly average mean temperature and total monthly precipitation for 2014**.

The study was conducted in 2014 on plants of *Pinus halepensis* Mill. and *Arbutus unedo* L. co-occurring at a site at Quisisana, Castellammare di Stabia (Naples) in southern Italy. The sampling site (40′683 N, 14′481 E, 346 m a. s. l) is characterized by typical Macchia vegetation, with shrub and tree species including *Quercus ilex* L., *P. pinaster* Aiton, *P. halepensis* Mill., *Castanea sativa* Mill., *Fraxinus ornus* L., *Acer opalus* Mill. subsp. *neapolitanum*, *Erica arborea* L., *Laburnum anagyroides* Medik, *A. unedo* L., and *Ruscus aculeatus* L. The climate at the site is Mediterranean, with hot, dry summers followed by mild, wet winters. According to data recorded at the closest meteorological station, 10 km from the sampling site (Pimonte, 40′672N, 14′50E, 370 m a. s. l), during the year of sampling 2014, annual mean temperature was 15.5°C with the hottest month being August (monthly average mean temperature of 23.3°C) and the coldest month being January (monthly average mean temperature of 9.8°C). The cumulative annual precipitation was 1020 mm; the wettest month was January, with a cumulative monthly precipitation of 447 mm, while the lowest value was reached in August (cumulative precipitation of 1.4 mm). The worst aridity period lasted from June to the beginning of September (**Figure 1**). Longer meteorological series are not available for the Pimonte site. Meteorological series from other nearby stations in the Campania region were analyzed and showed that meteorological conditions in 2014 did not deviate considerably from those registered in the period 2005–2014.

### Tree-Ring Data

Tree-ring chronologies were built through common dendroecological techniques to facilitate the synchronization of tree rings and IADFs in the collected microcores.

Core sampling was carried out in March 2015 on 15 dominant trees of *P. halepensis* and 15 plants of *A. unedo*. Diameter at breast height (DBH) was measured and two cores were taken at breast height as well from each tree (west and east directions) with an increment borer (diameter 5 mm). The cores were transported to the laboratory and air dried. The surface of the cores was polished using sand paper of different grain-sizes, and tree-ring width (TRW) measurements were made at a resolution of 0.01 mm, using LINTAB measurement equipment fitted with a stereoscope and equipped with TSAP Win software (Frank Rinn, Heidelberg, Germany). Tree-ring series were visually cross-dated and compared using standard dendrochronological techniques (Stokes and Smiley, 1968). The cross-dating accuracy was then checked using the program COFECHA (Holmes, 1983). The program ARSTAN (Cook, 1985) was used to remove growth trends related to tree age and competition, producing standardized tree-growth indices. Series were detrended with a 10-year spline to remove long-term growth trends embedded in the raw tree-ring series, which were thought to be induced by non-climatic influences, such as aging and competition between trees (Fritts, 1976). Once all series had been validated, tree-ring chronologies were constructed. Descriptive statistics were computed, including standard deviation (SD), which estimates the variability of measurements and the expressed population signal (EPS), thus indicating the level of coherence of the constructed chronology and how it portrays a hypothetical perfect population chronology.

The occurrence of IADFs was quantified in each core by considering each ring as a growth increment and distinguishing the true annual rings from IADFs through visual analysis of the features of boundaries (e.g., abruptness of changes between earlywood and latewood cells) and considering data from cross dating in cases of doubt (Cherubini et al., 2003; De Micco et al., 2016a). We finally calculated the frequency of IADFs in each plant as the ratio between the number of IADFs and the total number of increment growth. The Chi-square test was used to compare the occurrence of IADFs between the two species (two-way contingency table).

### Microcore Sampling and Microscopy

Microcores (1.8 mm in diameter) were collected with a Trephor tool (Rossi et al., 2006) from six trees of *P. halepensis* and eight plants of *A. unedo* at breast height, following a spiral with a distance of 2 cm between consecutive samples. Since we were interested in following xylogenesis throughout the aridity period, microcores were collected at weekly intervals starting from June. Sampling was interrupted in August because the observation of microcores (as reported below) showed that IADFs had been already formed. Further microcore collections were done after rain events at the beginning of September 2014 and at the end of the calendar year to check whether other IADFs had been formed in August or during fall.

Microcores were immediately fixed in 70% ethanol and stored at 4°C. Microcores were then embedded in paraffin using a Leica TP 1020-1 (Nussloch, Germany) tissue processor for dehydration in alcohol series (70, 90, 95, and 100%) and bio-clear (d-limonene) for paraffin infiltration. Paraffin blocks were cut with a semi-automatic rotary microtome RM 2245, Leica, (Nussloch), thus obtaining cross sections (9 μm thick), which were flattened on slides pre-treated with albumin. The slides were dried at 70°C for 30 min and cleaned of residual paraffin by washing with bio-clear and ethanol. The sections were then stained with a water solution of safranin and astra blue (Werf van der et al., 2007) and permanently mounted on glass slides in Euparal (Bioquip Rancho Dominguez, California). The sections were observed under a BX61 transmission light microscope (Olympus, Hamburg, Germany), while the quantification of anatomical parameters was performed through a Nikon Eclipse 800 microscope equipped with Nis Elements BR3 (Melville, NY, USA) image analysis software on microphotographs captured with a Nikon DS-Fi1 digital camera.

**FIGURE 2:**
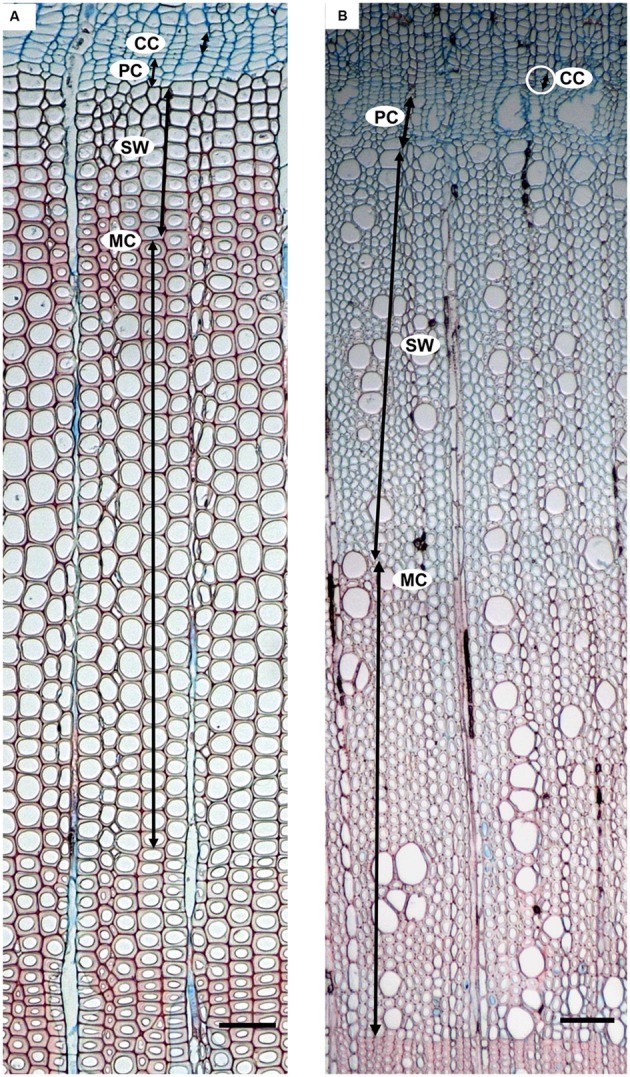
**Developing xylem in *Pinus halepensis***(A)** and *Arbutus unedo* (B)**. Moving from the cambial zone toward the center of the stem, the following cells are encountered: cambial cells (CC), enlarging post cambial cells (PC), cells developing secondary walls (SW), and mature cells with lignified secondary wall (MC). Scale bars = 100μm.

In *P. halepensis*, we focused on the development of tracheids, using visual criteria based on lumen size and wall thickness (de Luis et al., 2007, 2011a; Novak et al., 2016) measured by means of the eyepiece micrometer while looking through the microscope. The distinction between earlywood and latewood was based on the application of Mork’s definition (Mork, 1928). In *A. unedo* we focused on the development of vessels and imperforate tracheary elements, using visual criteria based not only on lumen size and wall thickness but also on vessel frequency; the transition between earlywood and latewood was often diffuse but latewood could be distinguished by the presence of narrower (halved lumen diameter) and less frequent vessels than earlywood (De Micco et al., 2016b). The following phases of cell development were considered according to de Luis et al. (2007) and Čufar et al. (2008, 2011): cambial cells (CC), post cambial cells (PC), cells with developing secondary wall (SW) and mature cells with lignified secondary wall (MC) (**Figure 2**). CC were radially flattened, with thin cell walls that stained blue. PC were enlarging cells in the phase of postcambial growth, which also stained blue. SW were immature xylem derivatives with developing (thickening and lignifying) secondary walls. SW cells showed birefringence under polarized light and stained blue and light red, depending on the progress of lignification. MC were cells without any trace of protoplast in the lumen and had fully deposited and lignified cell walls that colored intense red by safranin. The cambium was considered productive when PC were detected.

In *P. halepensis*, the number of cells in different developmental phases was counted. In *A. unedo*, since xylem consisted of conduits and fibers that were not arranged in ordered radial rows, the width of the cambium zone and developing xylem corresponding to the various developmental stages was measured as in Čufar et al. (2008, 2011). Measurements were taken in the cambium and in the developing xylem ring along three radial rows. Measurements along three radial rows were averaged.

Finally, in order to detect the time of IADF formation, we analyzed the microsections from subsequent microcores by focusing on cells in SW and MC phases to highlight changes in cell lumen size and wall thickness marking the transition from earlywood to latewood and vice versa. For each series of microcores, we classified SW and MC cells into four categories: earlywood (EW), latewood (LW), earlywood-like (EW-like), and secondary production of latewood (SLW) to distinguish them from customary LW. When the transition from earlywood to latewood was detected only once, and latewood formation was maintained until the end of the calendar year, the ring was classified as “not having an IADF”. In contrast, when the transition from earlywood to latewood was followed by additional transition from latewood to earlywood (EW-like) and from EW-like to latewood (SLW), then the ring was classified as “having an IADF”. We calculated the percent of plants showing SW and MC cells in each category per each date.

## Results

### Tree-Ring Chronologies and IADF Occurrence

**FIGURE 3:**
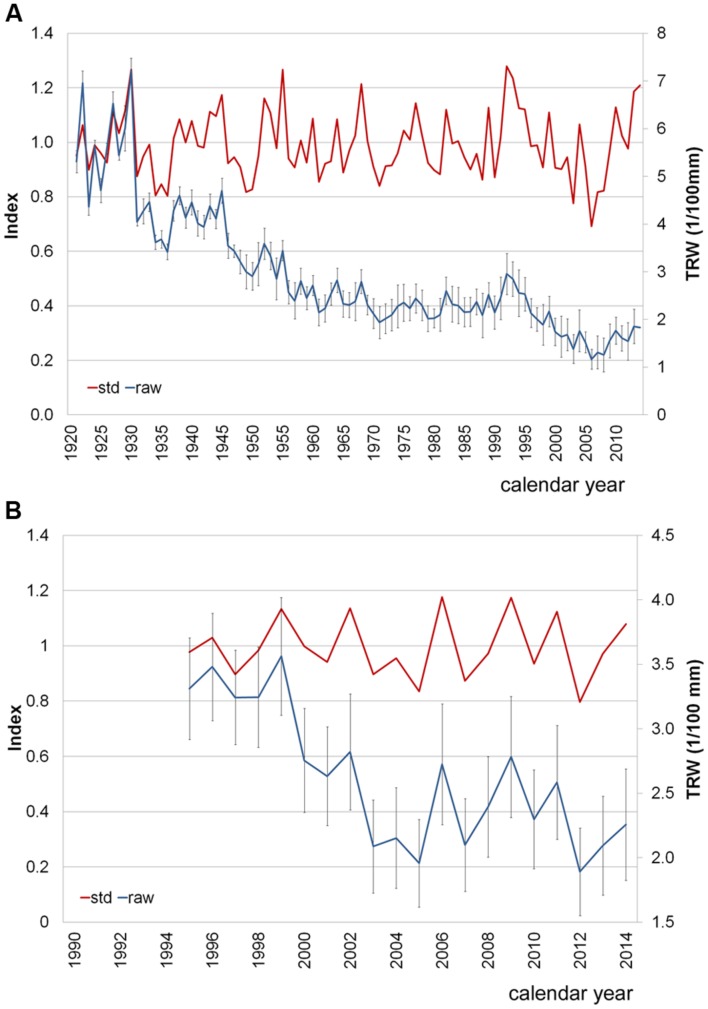
**Average ring-width chronologies using either raw (raw, blue line) or detrended data (std, standardized data, red line), in *P. halepensis***(A)** and *A. unedo* (B).** Standard deviation (SD) is shown for raw data.

**FIGURE 4:**
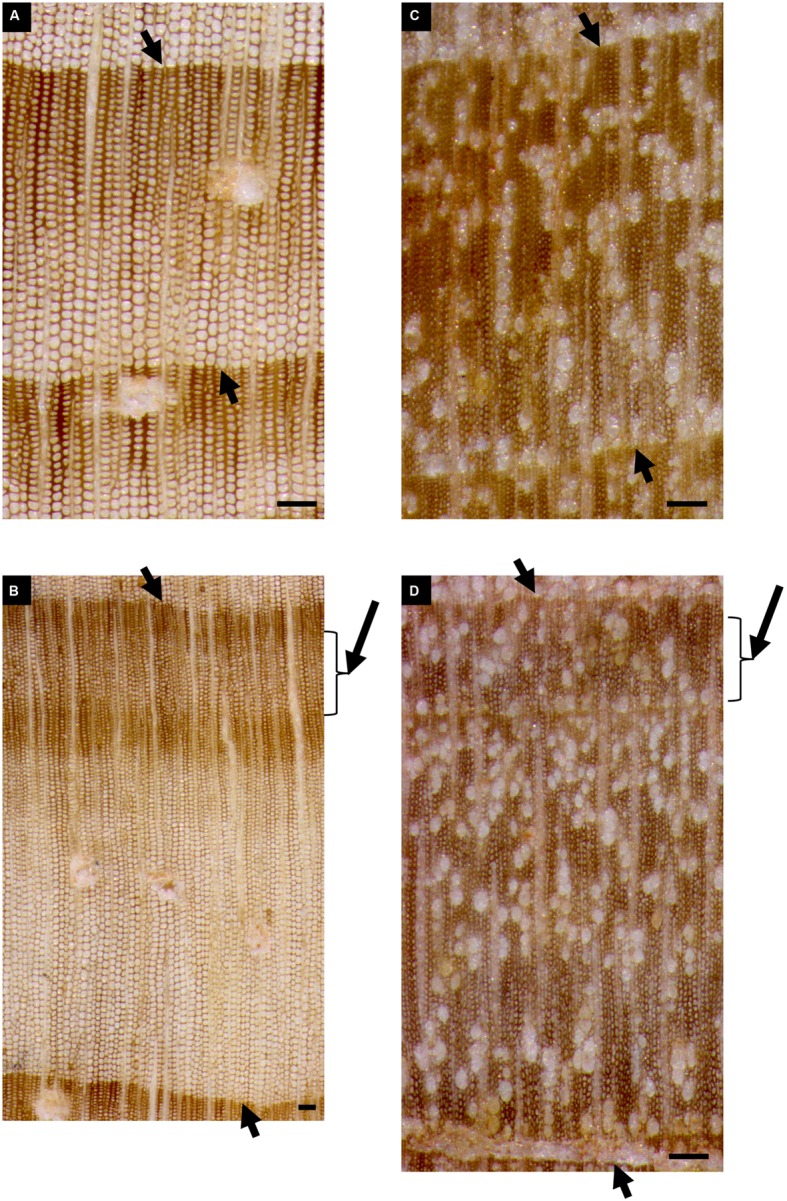
**Reflected light microscopy view of the tree ring corresponding to the 2014 calendar year in cores of *P. halepensis* and *A. unedo* with and without IADFs.** Tree rings of: *P. halepensis* without IADF **(A)**, *P. halepensis* with IADF **(B)**, *A. unedo* without IADF **(C)**, *A. unedo* with IADF **(D)**. Short arrows point to tree-ring boundaries; long arrows indicate the IADF. Bars = 100 μm.

The *P. halepensis* trees had a DBH of 52.24 ± 5.41 cm (mean value ± SD) and belonged to the same age class, with a mean of 90 ± 12 years (**Figure 3A**). High EPS values (>0.85) for the period of 1921–2014 indicated that the mean chronology was representative of radial growth variations of the whole population of trees (Wigley et al., 1984). The MS value (0.25) and the *r* bar value (0.82) showed a strong common growth signal among individuals. Thus, the variability among individuals and between twin cores from the same tree was not high. Despite the occurrence of IADFs, it was still possible to recognize, measure and cross-date the rings and build a robust mean chronology (**Figure 3A**). The percentage of IADFs in *P. halepensis* was 20.19 ± 2.63% (mean value ± SE), with a minimum of 6.19% and a maximum of 34.56%. In 2014, 58.3% of the trees formed IADFs appearing as earlywood-like cells within latewood (**Figures 4A,B**).

A different situation was found in the *A. unedo* plants. They were younger than the *P. halepensis* trees, with DBH of 7.97 ± 1.78 cm and related tree-ring chronologies spanning from 1995 to 2014 (**Figure 3B**). Very high variability was found among individuals and between twin cores from the same plant, as shown by high values of standard deviation (**Figure 3B**). Furthermore, the *A. unedo* cores contained a very high frequency of IADFs, hampering cross-dating. In this shrub hardwood, the percentage of IADFs was 36.65 ± 3.02% (mean value ± SE), with a minimum of 21.43 and maximum of 48.39%. In some cases, the tree-ring from the same calendar year contained more than one IADF. In 2014, 72.3% of the plants formed IADFs appearing as earlywood-like cells within latewood (**Figures 4C,D**).

The analysis of the two-way contingency table showed that the percentage of IADFs was significantly higher (*p* < 0.0001) in *A. unedo* than in *P. halepensis* tree-ring series.

### Cambial Productivity

**FIGURE 5:**
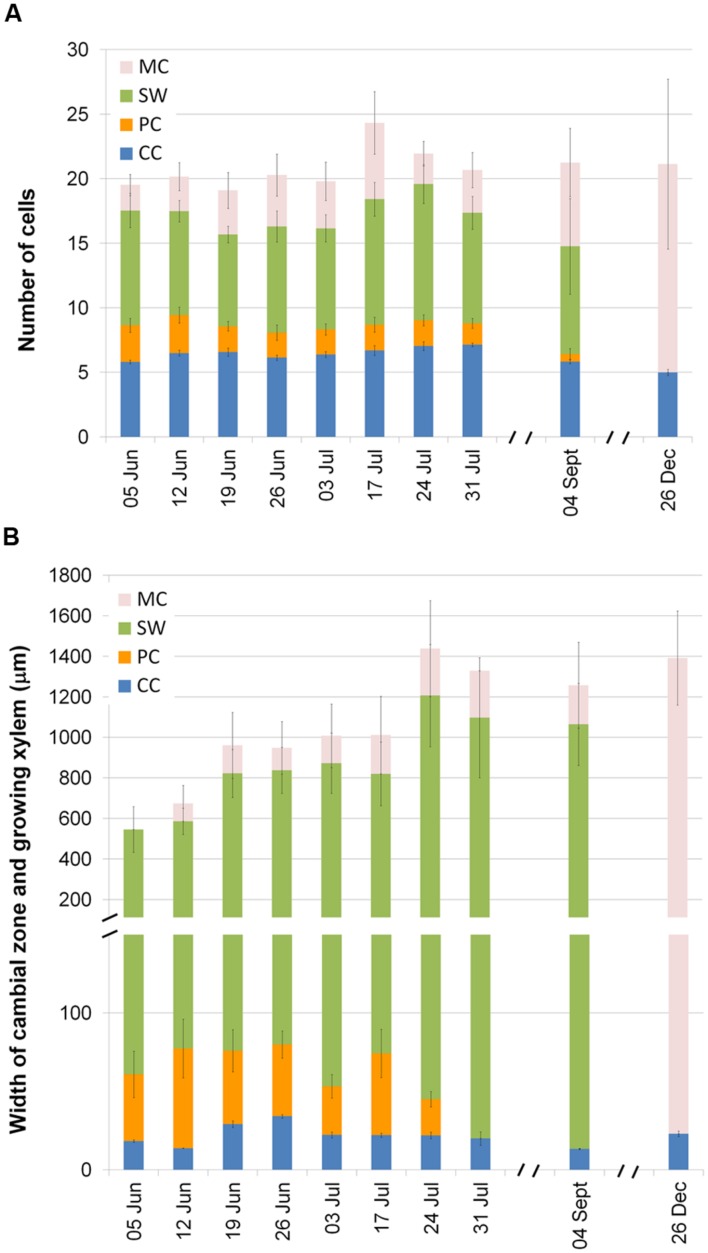
**Number of cells in various phases of xylem formation in 6 trees of *P. halepensis***(A)** and width of different developmental xylem zones in five plants of *A. unedo***(B)**: cambial cells (CC), enlarging post cambial cells (PC), cells developing secondary walls (SW), and mature cells with lignified secondary wall (MT).** Data from rings both forming and not forming IADFs in 2014 were considered together. Mean values and standard errors are shown.

As expected, xylem formation in *P. halepensis* and *A. unedo* had started prior to the first sampling on 5 June. At the beginning of June, in *P. halepensis* the cambial zone (CC) consisted of 5.81 ± 0.14 cells (mean value ± SE) and the current xylem growth ring consisted of 11.72 ± 1.65 cells in different phases of differentiation (PC and SW) and included 1.99 ± 0.81 fully differentiated cells (MC). The number of CC slightly increased in July (**Figure 5A**). The number of PC was highest on 12 June, remained more or less stable until the end of July and was very low at the beginning of September. The number of SW and MC cells varied due to the usual variability around the stem. In December, CC consisted of 5.00 ± 0.23 cells and no differentiating PC or SW cells were observed, so the current tree-ring consisted of MC cells only.

At the time of the first sampling, the CC of *A. unedo* was 18.35 ± 0.82 μm wide (mean value ± SE) and on average consisted of three cells (**Figure 5B**). Its width slightly increased thereafter and reached a maximum on 26 June (34.10 ± 1.10 μm). The thickness of the PC zone reached its maximum on 12 June (63.57 ± 18.67 μm), whereas almost no PC cells were observed on 31 July or at the beginning of September. The zone of SW cells remained wide throughout the summer. In September, the current tree-ring mainly consisted of SW with a small proportion of MC cells. At the end of December, we could observe no cell production and almost all cells of the current ring were fully differentiated. The width of the currently formed tree ring also varied around the stem in *A. unedo*.

### Wood Formation and IADFs

The overall analysis of the microcore data showed that at the time of first sampling, only 33.4 ± 4.9% (mean value standard error) and 46.5 ± 6.2% of the xylem increment (tree ring) of the current year had been already formed in *P. halepensis* and *A. unedo*, respectively. In June, it consisted of mainly SW cells with earlywood characteristics.

**FIGURE 6:**
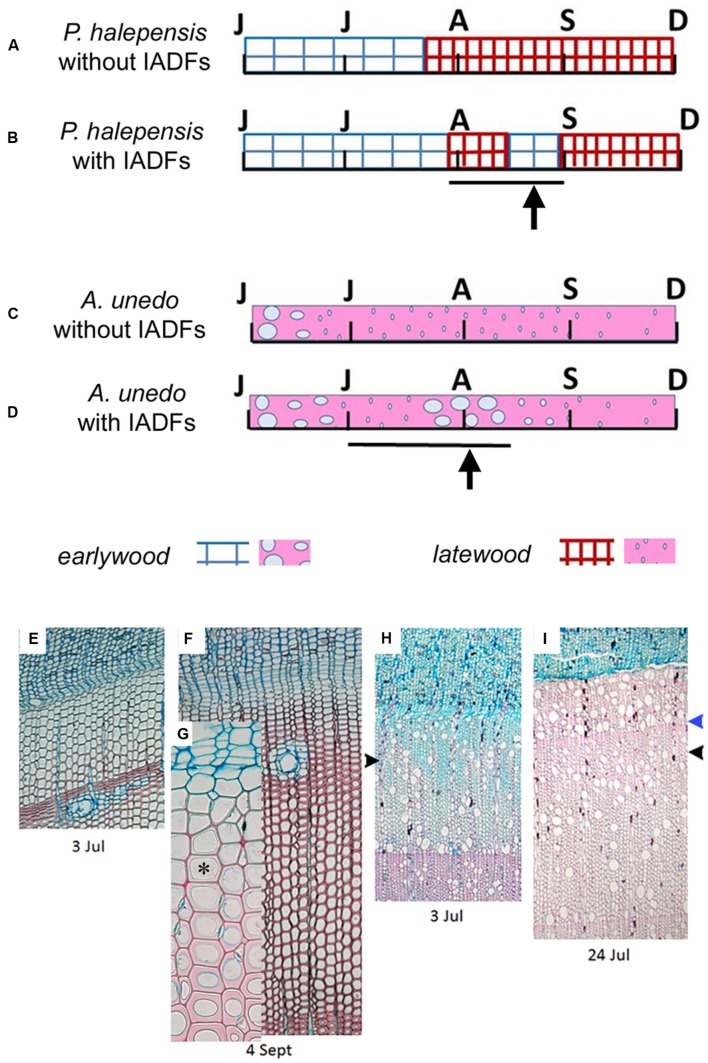
**(A–D)** Schematic qualitative presentation of different types of developing xylem in tree rings of *P. halepensis* **(A,B)** and *A. unedo* **(C,D)** with and without IADFs. Letters correspond to the months from June to December. Bar and arrow indicate the IADF position. **(E–I)** Light microscopy view of microsections of microcores of *P. halepensis* and *A. unedo* plants forming IADFs in the 2014 tree ring. E. *P. halepensis* increment growth consisting of cells in different phases of differentiation and MC of the sole earlywood-type. **(F–G)** *P. halepensis* increment growth with a band of latewood and first rows of maturing earlywood-like cells; asterisk indicates an earlywood-like tracheid with lignifying secondary cell wall (light red staining due to safranin) and lumen larger than the previously formed mature latewood-like tracheids. **(H)** *A. unedo* increment growth consisting of cells in different phases of differentiation and MC of the earlywood-type cells and first rows of latewood vessels (black arrowhead). **(I)** *A. unedo* increment growth with a band of latewood cells detected starting from 3 July (black arrowhead) and a band of earlywood-like cells detected starting from 17 July (blue arrowhead).

The analysis of anatomical characteristics of wood formed during summer 2014 was much easier in *P. halepensis* than in *A. unedo*. In *P. halepensis*, two (out of six) analyzed trees, had a “normal” 2014 tree ring, consisting of earlywood followed by latewood with no IADFs (**Figures 4A** and **6A**). In the other four trees, IADFs were formed during the summer (**Figures 4B** and **6B**). As summarized in **Table 1**, in the two trees without 2014-IADF, MC in the increment growth consisted of earlywood tracheids until mid-July (**Figures 4A and 6A,E**). The first latewood tracheids appeared on 24 July and the production of latewood tracheids continued until the completion of the annual ring (**Table 1**). In the other four trees, the first latewood tracheids appeared completed between 24 and 31 July, and latewood production was followed again by earlywood-like cells, with large lumina and thin cell walls on the successive dates. In samples collected in September, the first mature earlywood-like tracheids were observed (**Figures 6F,G**; **Table 1**) while current PC and SW cells evolved into latewood cells, as found in the last date analyzed (**Figures 4B** and **6B,F**; **Table 1**). From mid-September until the end of the year, only latewood cells were produced. It is worth highlighting that if IADFs formed in 2014 were classified by visual analysis of the complete tree ring without considering the timing of cambial activity, they could have been classified as either *E*-type IADF (latewood-like cells within earlywood) or *L*-type IADF (earlywood-like cells within latewood). By adding the information on xylogenesis, as well as temperature and precipitation of the corresponding calendar year, the rings with IADFs would be classified as *L*-IADFs.

**Table 1:** Percent of *Pinus halepensis* (PIHA) and *Arbutus unedo* (ARUN) plants whose cells in SW and MC phases were classified as earlywood (EW), latewood (LW), earlywood-like (EW-like), or secondary latewood production (SLW) in the 2014-tree rings without (No) or with (Yes) IADFs, per each analyzed week (day of the year is reported in parenthesis below the date).

			Percent of plants producing specific wood type (%)										
Species	IADF	SW-MC	05 June (156)	12 June (163)	19 June (170)	26 June (177)	03 July (184)	10 July (191)	17 July (198)	24 July (205)	31 July (212)	04 September (247)	26 December (360)
*PIHA*	No	EW	100	100	100	100	100	100	100	0	0	0	0
		LW	0	0	0	0	0	0	0	100	100	100	100
		EW-like	0	0	0	0	0	0	0	0	0	0	0
		SLW	0	0	0	0	0	0	0	0	0	0	0
*PIHA*	Yes	EW	100	100	100	100	100	100	100	75	0	0	0
		LW	0	0	0	0	0	0	0	25	100	0	0
		EW-like	0	0	0	0	0	0	0	0	0	100	0
		SLW	0	0	0	0	0	0	0	0	0	0	100
*ARUN*	No	EW	100	50	0	0	0	0	0	0	0	0	0
		LW	0	50	100	100	100	100	100	100	100	100	100
		EW-like	0	0	0	0	0	0	0	0	0	0	0
		SLW	0	0	0	0	0	0	0	0	0	0	0
*ARUN*	Yes	EW	100	100	75	50	25	25	0	0	0	0	0
		LW	0	0	25	50	75	75	75	50	25	0	0
		EW-like	0	0	0	0	0	0	25	25	25	25	0
		SLW	0	0	0	0	0	0	0	25	50	75	100

In the case of *A. unedo*, the formation of the different types of wood showed high variability among plants. It was possible to follow the genesis of different wood structures in six (out of the eight) analyzed plants. More specifically, in the other two plants, consecutive microcores (taken in spiral along the stem) showed very different growth increments. Due to the great variability of ring width around the stem it was not always possible to differentiate unequivocally between false and very narrow “normal” tree-rings. These plants were also characterized by very diverse twin cores and corresponding tree-ring series, which could not be cross-dated. We therefore excluded them from the analysis. Of the remaining six plants, two did not form IADFs. As summarized in **Table 1**, in these plants, the first mature latewood vessels were evident on 12 and 19 June and the production of this cell type continued until the completion of the annual ring (**Figures 4C and 6C**; **Table 1**). In the other four plants, MC observed until the middle of June were of the earlywood type (**Figure 6C**; **Table 1**). The first mature latewood vessels appeared progressively from 19 June to 17 July in the different plants (**Figures 6H,I**, black arrowhead, **Table 1**). In these four plants, after the formation of the first latewood vessels, new earlywood-like cells were formed starting from the middle of July (**Figures 6D,I** blue arrowhead, **Table 1**). A successive production of very narrow latewood was recorded progressively starting from 24 July. Similar to *P. halepensis*, also in *A. unedo* we classified the 2014-IADFs as *L*-IADFs. From September until the end of the year, only latewood cells were produced and no other IADFs were formed. Only in one plant did we finally record two *L*-IADFs in the 2014 ring.

## Discussion

Analysis of cambial activity supported by dendro-anatomical investigations in the softwood *P. halepensis* and the hardwood *A. unedo* allowed us to reconstruct the timing of IADF formation and to hypothesize possible reasons for their formation.

Intra-annual density fluctuation identification and classification is still mainly done by visual analysis of cores and/or microsections under the microscope. When analyzing a tree ring for the presence of an IADF, the operator considers the sequence of wood types (earlywood, latewood) along the tree-ring width. A calendar tree-ring contains an IADF when the sequence of wood types encountered from the beginning of the tree ring toward the cambium is the following: earlywood, latewood (or latewood-like cells), earlywood (or earlywood-like cells) and latewood. The IADF can then be classified into *E*- or *L*-type IADF (De Micco et al., 2016a). It is clear that the classification into one type or the other depends on identification of the region of the ring in which the true latewood begins. Analysis of relations between IADFs and climatic data also helps their classification because the two types of IADFs, can occur at different positions within the growth ring and have completely different functional significance. For example, *A. unedo* growing at a site in Southern Italy has been recently shown to be able to form two types of IADFs, according to water availability at the growing sites (Battipaglia et al., 2010, 2014). More specifically, under xeric conditions, this species forms *E*-IADFs, meaning that the “anomaly” is the formation of latewood-like cells in a period when earlywood is expected, thus triggered by a particularly severe or unexpected period of drought (Battipaglia et al., 2010). Under more mesic conditions, *A. unedo* developed *L*-IADFs, meaning that the “anomaly” was the formation of earlywood-like cells in a period when latewood should have been formed; such *L*-IADFs were ascribed to rain events occurring during fall after a period of summer drought. This mechanism of *L*-IADF formation has also been highlighted as typical of *Pinus* species (Zalloni et al., 2016). Such a seasonal dynamics of wood development follows the bimodal pattern described for *P. halepensis* from arid and semi-arid ecosystems in Spain (de Luis et al., 2011a), and also reported recently by Camarero et al. (2010) in *Juniperus thurifera* from sub-humid and semi-arid Mediterranean continental sites in Spain, and in the co-occurring *P. halepensis* only in the more xeric site. Such a pattern has been also reported for *P. pinaster* growing on sand dunes in Portugal by Vieira et al. (2015). The bimodal pattern of wood growth is due to cambial reactivation triggered by spring and fall precipitations, which control cell enlargement and cell wall deposition after winter low-temperatures and summer drought stresses (Camarero et al., 2010; Pacheco et al., 2016). In our study, to classify IADFs objectively in the two analyzed species and to evaluate the period of their formation, xylogenesis proved to be a valuable tool because it allowed identification of the starting moment for latewood formation, as well as observation of the progressive formation of IADFs while they were “under construction” (de Luis et al., 2011b; Novak et al., 2016). Considering the timing of IADF formation and keeping in mind the precipitation pattern observed in 2014, we confidently classified the 2014 IADFs as *L*-type, following a bimodal pattern of xylogenesis. However, in our case, the second growth flash, leading to the second earlywood formation during the calendar year, already occurred in summer and not in fall as hypothesized for Mediterranean species (De Micco et al., 2016a). We could speculate that, in this specific case, fall precipitations can lead to more than one IADF per ring. This is sometimes observed in *A. unedo* where such additional IADFs would be better classified *L^+^*-IADFs according to Campelo et al. (2007b, 2013). The high variability of behavior of different plants in various environments strengthen the need for a common and unambiguous classification of IADFs, also considering their position within tree rings, to achieve a correct functional interpretation of them (De Micco et al., 2016a).

In the two analyzed species, the formation of IADFs appeared irregularly in time and different individuals showed different predispositions to IADF development in different years, in agreement with other studies (Cherubini et al., 2003). This phenomenon indicates the capacity of these two species to switch from a unimodal pattern of xylogenesis toward a bimodal wood growth, and vice versa, from one year to another. The two species responded with the same strategy (i.e., the formation of *L*-IADFs) to fluctuating environmental conditions, but their sensitivity was different. The onset of latewood formation and the appearance of IADFs was shifted a couple of weeks earlier in *A. unedo* than in *P. halepensis*. However, the occurrence of mature latewood cells in *A. unedo* was still delayed compared with the few studied broadleaved deciduous species such as chestnut and beech (Čufar et al., 2008, 2011). *A. unedo* appeared to be more sensitive than *P. halepensis* also because of a higher frequency of IADFs and because several growth rings were often formed per year. This would indicate a better and quicker ability to induce dormancy and activation of the cambium after fluctuating environmental conditions. This different sensitivity is not surprising because of the different size, age, and growth and reproductive strategies of the two species which dictate different hydraulic constraints and resource use/allocation. For instance the younger age of *A. unedo* plants compared with *P. halepensis* can partly explain their higher tendency to form IADFs. Indeed, IADF frequency is generally higher in younger than in older trees, probably not only because young plants have shallow root systems and may thus be less able to access deep soil layers but also because young plants show increased tree-ring width, which is positively correlated to IADF frequency (Rigling et al., 2001, 2002; Cherubini et al., 2003; Bogino and Bravo, 2009; Hoffer and Tardif, 2009; Rozas et al., 2011; Novak et al., 2013b; Campelo et al., 2015; Pacheco et al., 2016).

The appearance of the first mature latewood conduits in the two analyzed species occurred well after the onset of the summer drought period (May). Indeed, wood formation is the result of the integration of complex cellular processes, in which cell-wall thickening and lignification lags behind cell enlargement by as much as a month or more, according to the recent model by Cuny et al. (2015). The time-lag observed between the increase in xylem size (linked to cell enlargement) and accumulation of woody biomass (due to cell wall thickening and lignification) shows differences between earlywood and latewood, and it has been quantified in a range of 27–49 days in different climates, with maximum values in the Mediterranean region (Cuny et al., 2014, 2015). The application of this time-lag principle to our samples would indicate that latewood conduits are produced by cambium activity from the beginning of the drought period but, given that cell-wall deposition and maturation requires several weeks (up to ≈ 7) (Prislan et al., 2009, 2013a; Cuny et al., 2014), latewood thick-walled cells become evident only later. Similarly, in tree rings showing IADFs, a new production of earlywood may have been primed by rain pulse events during July (which was characterized by almost twice as much precipitation as June). Increased cell enlargement in July thus possibly triggered the formation of earlywood-like cells, which appeared mature only at the end of July and August, based on the time required for cell wall thickening (up to≈ 4 weeks) as estimated by Prislan et al. (2009, 2013b) and Cuny et al. (2014).

The two analyzed species showed wood production during the summer drought period, which is considered limiting for growth, also perhaps inducing a halt in cambial productivity in some species (Liang et al., 2006; Camarero et al., 2010; Pacheco et al., 2016). However, this could be explained by temporary favorable conditions, specific resource use efficiency and strategies, or might have another basis, phylogenetic or biogeographical, given that many Mediterranean species perform costly metabolic processes precisely under unfavorable summer drought periods (Aronne and Wilcock, 1994).

From a methodological viewpoint, the micro-coring technique and analysis of xylogenesis were easily applied to tree rings showing IADFs of *P. halepensis*, whereas it was more difficult to apply them in *A. unedo* because of the very high variability of IADFs among and within plants in different years. However, cross-dating was a helpful tool for identifying the beginning of the last growth increment (2014) and for verifying wood growth variability in the same stem. We thus suggest that, when studying xylogenesis in Mediterranean plants forming a high frequency of IADFs and more than one IADF per calendar year, it is useful to perform a preliminary tree-ring analysis in order to predict the applicability of micro-coring in single plants, thus excluding from the micro-coring experiment those trees having high variability of wood growth around the stem and those having clear anomalies.

To conclude, with the support of dendroecological analysis, micro-coring allowed the identification of the period of IADF formation in the two Mediterranean species confirming the hypothesis of the occurrence of a bimodal pattern of cambial activity. Both species were prone to form IADFs that were classified as *L*-type, indicating a period of growth flash due to favorable environmental conditions for growth occurring during summer, and not in fall as reported for other Mediterranean species (e.g., Camarero et al., 2010; de Luis et al., 2011b; Campelo et al., 2013; Carvalho et al., 2015; Zalloni et al., 2016). A possible explanation, still to be verified is that different kinds of *L*-IADFs exist (*L*- and *L*^+^-type); they would be triggered by temporary favorable conditions occurring during summer and fall, respectively.

The formation of *L*-IADFs can be considered a way of improving the hydraulic conductivity of wood (Sperry et al., 2006) when water is unexpectedly available after a period of severe drought. As a consequence, species showing high plasticity in cambial productivity, thus prone to form *L*-IADFs, promptly after a positive climatic event (e.g., unexpected summer rain pulse) following a period of severe drought (e.g., dry periods at the end of spring), should have an advantage under fluctuating environmental conditions over those not able to form IADFs.

## Author Contributions

VDM, AB and GB made a substantial contribution to the conception and design of the study. AB performed sample collection. AB and MM performed sample preparation. VDM, AB, JG, KC and GB made a substantial contribution to the analysis of tree-ring series and anatomical signals in microsections and in data analysis. VDM, AB, KC and GA contributed to the interpretation of the overall data. VDM, KC, MM, GA and GB contributed to the analysis tools. VDM wrote the main part of the manuscript. AB and GB contributed to writing the text. All authors contributed to final revisions of the manuscript and read and approved the submitted version of the manuscript.

## Conflict of Interest Statement

The authors declare that the research was conducted in the absence of any commercial or financial relationships that could be construed as a potential conflict of interest.

## References

[B1] Aronne G. Wilcock C. C. (1994). Reproductive characteristics and breeding system of shrubs of the mediterranean region. *Funct. Ecol.* 8 69–76. 10.2307/2390113

[B2] Battipaglia G. Campelo F. Vieira J. Grabner M. De Micco V. Nabais C. (2016). Structure and function of intra-annual density fluctuation: mind the gaps. *Front. Plant Sci.* 7:595 10.3389/fpls.2016.00595 PMC4858752 27200063

[B3] Battipaglia G. De Micco V. Brand W. A. Linke P. Aronne G. Saurer M. (2010). Variations of vessel diameter and δ13C in false rings of *Arbutus unedo* L. reflect different environmental conditions. *New Phytol.* 188 1099–1112. 10.1111/j.1469-8137.2010.03443.x 20840507

[B4] Battipaglia G. De Micco V. Brand W. A. Saurer M. Aronne G. Linke P. (2014). Drought impact on water use efficiency and intra-annual density fluctuations in *Erica arborea* on Elba (Italy). *Plant Cell Environ.* 37 382–391. 10.1111/pce.12160 23848555

[B5] Beeckman H. (2016). Wood anatomy and trait-based ecology. *IAWA J.* 37 127–151. 10.1163/22941932-20160127

[B6] Bogino S. Bravo F. (2009). Climate and intra-annual density fluctuations in *Pinus pinaster* subsp. mesogeensis in Spanish woodlands. *Can. J. For. Res.* 39 1557–1565. 10.1139/x09-074

[B7] Camarero J. J. Olano J. M. Parras A. (2010). Plastic bimodal xylogenesis in conifers from continental Mediterranean climates. *New Phytol.* 185 471–480. 10.1111/j.1469-8137.2009.03073.x 19895415

[B8] Campelo F. Gutičrrez E. Ribas M. Nabais C. Freitas H. (2007a). Relationships between climate and double rings in *Quercus ilex* from northeast Spain. *Can. J. For. Res* 37 1915–1923. 10.1139/X07-050

[B9] Campelo F. Nabais C. Freitas H. Gutičrrez E. (2007b). Climatic significance of tree-ring width and intra-annual density fluctuations in *Pinus pinea* from a dry Mediterranean area in Portugal. *Ann. For. Sci.* 64 229–238. 10.1051/forest:2006107

[B10] Campelo F. Nabais C. Gutičrrez E. Freitas H. García-González I. (2010). Vessel features of *Quercus ilex* L. growing under Mediterranean climate have a better climatic signal than tree-ring width. *Trees* 24 463–470. 10.1007/s00468-010-0414-0

[B11] Campelo F. Vieira J. Battipaglia G. de Luis M. Nabais C. Freitas H. (2015). Which matters most for the formation of intra-annual density fluctuations in *Pinus pinaster*: age or size? *Trees* 29 237–245. 10.1007/s00468-014-1108-9

[B12] Campelo F. Vieira J. Nabais C. (2013). Tree-ring growth and intra-annual density fluctuations of *Pinus pinaster* responses to climate: does size matter? *Trees* 27 763–772. 10.1007/s00468-012-0831-3

[B13] Carvalho A. Nabais C. Vieira J. Rossi S. Campelo F. (2015). Plastic response of tracheids in *Pinus pinaster* in a water-limited environment: adjusting lumen size instead of wall thickness. *PLoS ONE* 10:e0136305 10.1371/journal.pone.0136305 PMC4549277 26305893

[B14] Cherubini P. Gartner B. L. Tognetti R. Bräker O. U. Schoch W. Innes J. L. (2003). Identification, measurement and interpretation of tree rings in woody species from mediterranean climates. *Biol. Rev.* 78 119–148. 10.1017/s1464793102006000 12620063

[B15] Cook E. R. (1985). *A Time-Series Analysis Approach to Tree-Ring Standardization.* Ph.D. Dissertation, University of Arizona, Tucson 175.

[B16] Čufar K. Cherubini M. Gričar J. Prislan P. Spina S. Romagnoli M. (2011). Xylem and phloem formation in chestnut (*Castanea sativa* Mill.) during the 2008 growing season. *Dendrochronologia* 29 127–134. 10.1016/j.dendro.2011.01.006

[B17] Čufar K. Prislan P. Gričar J. (2008). Cambial activity and wood formation in beech (*Fagus sylvatica*) during the 2006 growth season. *Wood Res.* 53 1–10.

[B18] Cuny H. E. Rathgeber C. B. K. Frank D. Fonti P. Fournier M. (2014). Kinetics of tracheid development explain conifer tree-ring structure. *New Phytol.* 203 1231–1241. 10.1111/nph.12871 24890661

[B19] Cuny H. E. Rathgeber C. B. K. Frank D. Fonti P. Mäkinen H. Prislan P. (2015). Woody biomass production lags stem-girth increase by over one month in coniferous forests. *Nat. Plants* 1:160 10.1038/nplants.2015.160 27251531

[B20] de Luis M. Gričar J. Čufar K. Raventos J. (2007). Seasonal dynamics of wood formation in *Pinus halepensis* from dry and semi-arid ecosystems in Spain. *IAWA J.* 28 389–404. 10.1163/22941932-90001651

[B21] de Luis M. Novak K. Raventós J. Gričar J. Prislan P. Čufar K. (2011a). Cambial activity, wood formation and sapling survival of *Pinus halepensis* exposed to different irrigation regimes. *For. Ecol. Manage.* 262 1630–1638. 10.1016/j.foreco.2011.07.013

[B22] de Luis M. Novak K. Raventós J. Gričar J. Prislan P. Čufar K. (2011b). Climate factors promoting intra-annual density fluctuations in Aleppo pine (*Pinus halepensis*) from semiarid sites. *Dendrochronologia* 29 163–169. 10.1016/j.dendro.2011.01.005

[B23] De Micco V. Aronne G. (2009). Seasonal dimorphism in wood anatomy of the Mediterranean *Cistus incanus* L. subsp. incanus. *Trees* 23 981–989. 10.1007/s00468-009-0340-1

[B24] De Micco V. Battipaglia G. Balzano A. Cherubini P. Aronne G. (2016b). Are wood fibres as sensitive to environmental conditions as vessels in tree rings with intra-annual density fluctuations (IADFs) in Mediterranean species? *Trees* 30 971–983. 10.1007/s00468-015-1338-5

[B25] De Micco V. Battipaglia G. Brand W. A. Linke P. Saurer M. Aronne G. (2012). Discrete versus continuous analysis of anatomical and δ13C variability in tree rings with intra-annual density fluctuations. *Trees* 26 513–524. 10.1007/s00468-011-0612-4

[B26] De Micco V. Battipaglia G. Cherubini P. Aronne G. (2014). Comparing methods to analyse anatomical features of tree rings with and without intra-annual density fluctuations (IADFs). *Dendrochronologia* 32 1–6. 10.1016/j.dendro.2013.06.001

[B27] De Micco V. Campelo F. de Luis M. Bräuning A. Grabner M. Battipaglia G. (2016a). Intra-annual density fluctuations in tree rings: how, when, where and why? *IAWA J.* 37 232–259.

[B28] De Micco V. Saurer M. Aronne G. Tognetti R. Cherubini P. (2007). Variations of wood anatomy and δ13C within-tree rings of coastal *Pinus pinaster* showing intra-annual density fluctuations. *IAWA J.* 28 61–74. 10.1163/22941932-90001619

[B29] Eckstein D. Liese W. Shigo A. L. (1979). Relationship of wood structure to compartmentalization of discoloured wood in hybrid poplar. *Can. J. For. Res.* 9 205–210. 10.1139/x79-036

[B30] Eckstein D. Schmidt B. (1974). Dendroklimatologische untersuchungen an stieleichen aus dem maritimen klimagebiet Schleswig-Holsteins. *Angew. Bot.* 48 371–383.

[B31] Esper J. Frank D. C. Timonen M. Zorita E. Wilson R. J. S. Luterbacher J. (2012). Orbital forcing of tree-ring data. *Nature Clim. Change* 2 862–866. 10.1038/nclimate1589

[B32] Fonti P. Von Arx G. Garcia-Gonzalez I. Eilmann B. Sass-Klaassen U. Gartner H. (2010). Studying global change through investigation of the plastic responses of xylem anatomy in tree rings. *New Phytol.* 185 42–53. 10.1111/j.1469-8137.2009.03030.x 19780986

[B33] Fritts H. C. (1976). *Tree Rings and Climate.* Caldwell, NJ: The Blackburn press.

[B34] Gričar J. Prislan P. Gryc V. Vavrčík H. de Luis M. Čufar K. (2014). Plastic and locally adapted phenology in cambial seasonality and production of xylem and phloem cells in *Picea abies* from temperate environments. *Tree Physiol.* 34 869–881. 10.1093/treephys/tpu026 24728295

[B35] Grudd H. (2008). Torneträsk tree-ring width and density ad 500–2004: a test of climatic sensitivity and a new 1500-year reconstruction of north Fennoscandian summers. *Clim. Dyn.* 31 843–857. 10.1007/s00382-007-0358-2

[B36] Hoffer M. Tardif J. C. (2009). False rings in jack pine and black spruce trees from eastern Manitoba as indicators of dry summers. *Can. J. For. Res.* 39 1722–1736. 10.1139/x09-088

[B37] Holmes R. L. (1983). Computer-assisted quality control in tree-ring dating and measurement. *Tree-Ring Bull.* 1983 51–67.

[B38] IPCC, Working Group I Stocker T. F. Qin D. Plattner G.-K. (2013). *Climate Change 2013: The Physical Science Basis. Contributionof Working Group I to the Fifth Assessment Report of the Intergovernmental Panel on Climate Change. IPCC AR5:1535.* New York, NY: Cambridge University Press.

[B39] Liang E. Shao X. Eckstein D. Huang L. Liu X. (2006). Topography- and species-dependent growth responses of *Sabina przewalskii* and *Picea crassifolia* to climate on the northeast Tibetan Plateau. *For. Ecol. Manage.* 236 268–277. 10.1016/j.foreco.2006.09.016

[B40] Mork E. (1928). Die qualität des fichtenholzes unter besonderer rücksichtnahme auf schleif-und papierholz. *Der Papier-Fabrikant* 26 741–747.

[B41] Novak K. de Luis M. Gričar J. Prislan P. Merela M. Smith K. T. (2016). Missing and dark rings associated with drought in *Pinus halepensis*. *IAWA J.* 37 260–274.

[B42] Novak K. de Luis M. Raventós J. Čufar K. (2013a). Climatic signals in tree-ring widths and wood structure of *Pinus halepensis* in contrasted environmental conditions. *Trees* 27 927–936. 10.1007/s00468-013-0845-5

[B43] Novak K. Sánchez M. A. S. Čufar K. Raventós J. de Luis M. (2013b). Age, climate and intra-annual density fluctuations in in Spain. *IAWA J.* 34 459–474. 10.1163/22941932-00000037

[B44] Pacheco A. Camarero J. J. Carrer M. (2016). Linking wood anatomy and xylogenesis allows pinpointing of climate and drought influences on growth of coexisting conifers in continental Mediterranean climate. *Tree Physiol.* 36 502–512. 10.1093/treephys/tpv125 26705312

[B45] Pérez-de-Lis G. Rossi S. Vázquez-Ruiz R. A. Rozas V. García-González I. (2016). Do changes in spring phenology affect earlywood vessels? Perspective from the xylogenesis monitoring of two sympatric ring-porous oaks. *New Phytol.* 209 521–530. 10.1111/nph.13610 26295692

[B46] Prislan P. Čufar K. Koch G. Schmitt U. Gričar J. (2013a). Review of cellular and subcellular changes in the cambium. *IAWA J.* 34 391–407. 10.1163/22941932-00000032

[B47] Prislan P. Gričar J. De Luis M. Smith K. T. Čufar K. (2013b). Phenological variation in xylem and phloem formation in *Fagus sylvatica* from two contrasting sites. *Agric. For. Meteorol.* 180 142–151. 10.1016/j.agrformet.2013.06.001

[B48] Prislan P. Koch G. Čufar K. Gričar J. Schmitt U. (2009). Topochemical investigations of cell walls in developing xylem of beech (*Fagus sylvatica* L.). *Holzforschung* 63 482–490. 10.1515/hf.2009.079

[B49] Rigling A. Bräker O. Schneiter G. Schweingruber F. (2002). Intra-annual tree-ring parameters indicating differences in drought stress of *Pinus sylvestris* forests within the Erico-Pinion in the Valais (Switzerland). *Plant Ecol.* 163 105–121. 10.1023/a:1020355407821

[B50] Rigling A. Waldner P. O. Forster T. Bräker O. U. Pouttu A. (2001). Ecological interpretation of tree-ring width and intraannual density fluctuations in *Pinus sylvestris* on dry sites in the central Alps and Siberia. *Can. J. For. Res.* 31 18–31. 10.1139/cjfr-31-1-18

[B51] Rossi S. Deslauriers A. Anfodillo T. Carrer M. (2008). Age-dependent xylogenesis in timberline conifers. *New Phytol.* 177 199–208. 10.1111/j.1469-8137.2007.02235.x 17944824

[B52] Rossi S. Deslauriers A. Anfodillo T. Morin H. Saracino A. Motta R. (2006). Conifers in cold environments synchronize maximum growth rate of tree-ring formation with day length. *New Phytol.* 170 301–310. 10.1111/j.1469-8137.2006.01660.x 16608455

[B53] Rossi S. Deslauriers A. Morin H. (2003). Application of the Gompertz equation for the study of xylem cell development. *Dendrochronologia* 21 33–39. 10.1078/1125-7865-00034

[B54] Rossi S. Morin H. Deslauriers A. (2012). Causes and correlations in cambium phenology: towards an integrated framework of xylogenesis. *J. Exp. Bot.* 63 2117–2126. 10.1093/jxb/err423 22174441 PMC3295399

[B55] Rozas V. García-González I. Zas R. (2011). Climatic control of intra-annual wood density fluctuations of *Pinus pinaster* in NW Spain. *Trees* 25 443–453. 10.1007/s00468-010-0519-5

[B56] Schweingruber F. H. (1978). *Mikroskopische Holzanatomie.* Birmensdorf: Eidgenossische Anstalt fur das forstliche Versuchswesen.

[B57] Sperry J. S. Hacke U. G. Pittermann J. (2006). Size and function in conifer tracheids and angiosperm vessels. *Am. J. Bot.* 93 1490–1500. 10.3732/ajb.93.10.1490 21642096

[B58] Stokes M. Smiley T. (1968). *An Introduction to Tree-Ring Dating. University of Chicago, Chicago, Reprinted 1996.* Tucson, AZ: University of Arizona Press.

[B59] Tardif J. (1996). “Earlywood, latewood and total ring width of a ringporous species (*Fraxinus nigra* Marsh) in relation to climatic and hydrologic factors,” in *Tree Rings, Environment and Humanity*, eds Dean J. S. Meko D. M. Swetnam T. W. (Tucson, AZ: Radiocarbon. Dept. of Geosciences, Univ. Arizona), 315–324.

[B60] Vaganov E. A. Huges M. K. Shashkin A. V. (2006). *Growth Dynamics of Conifer Tree Rings. Images of Past and Future Environments.* Berlin: Springer-Verlag.

[B61] Vieira J. Campelo F. Nabais C. (2010). Intra-annual density fluctuations of *Pinus pinaste*r are a record of climatic changes in the western Mediterranean region. *Can. J. For. Res.* 40 1567–1575. 10.1139/x10-096

[B62] Vieira J. Campelo F. Rossi S. Carvalho A. Freitas H. Nabais C. (2015). Adjustment capacity of maritime pine cambial activity in drought-prone environments. *PLoS ONE* 10:e0126223 10.1371/journal.pone.0126223 PMC4427410 25961843

[B63] Vieira J. Rossi S. Campelo F. Freitas H. Nabais C. (2014). Xylogenesis of *Pinus pinaster* under a Mediterranean climate. *Ann. For. Sci.* 71 71–80. 10.1007/s13595-013-0341-5

[B64] von Arx G. Carrer M. (2014). ROXAS – A new tool to build centuries-long tracheid-lumen chronologies in conifers. *Dendrochronologia* 32 290–293. 10.1016/j.dendro.2013.12.001

[B65] Werf van der G. W. Sass-Klaassen U. G. W. Mohren G. M. J. (2007). The impact of the 2003 summer drought on the intra-annual growth pattern of beech (*Fagus sylvatica* L.) and oak (*Quercus robur* L.) on a dry site in the Netherlands. *Dendrochronologia* 25 103–112. 10.1016/j.dendro.2007.03.004

[B66] Wigley T. M. L. Briffa K. R. Jones P. D. (1984). On the average value of correlated time series, with applications in dendroclimatology and hydrometeorology. *J. Clim. Appl. Meteorol.* 23 201–213. 10.1175/1520-0450(1984)023<0201:OTAVOC>2.0.CO;2

[B67] Xia J. Y. Niu S. L. Ciais P. Janssens I. A. Chen J. Q. Ammann C. (2015). Joint control of terrestrial gross primary productivity by plant phenology and physiology. *Proc. Nat. Acad. Sci.* 112 2788–2793. 10.1073/pnas.1413090112 25730847 PMC4352779

[B68] Zalloni E. De Luis M. Campelo F. Novak K. De Micco V. Di Filippo A. (2016). Regional patterns of intra-annual density fluctuations frequency in mediterranean pines. *Front. Plant Sci.* 7:579 10.3389/fpls.2016.00579 PMC4852653 27200052

